# Supplementary material for: Bioinspired trimesic acid anchored electrocatalysts with unique static and dynamic compatibility for enhanced water oxidation
Source: Nat Commun. 2023 Oct 23;14:6714. doi: 10.1038/s41467-023-42292-5 (PMC10593801; doi:10.1038/s41467-023-42292-5)
Supplement: Supplementary file 1 — Supplementary Information [file 41467_2023_42292_MOESM1_ESM.pdf]

Supplementary Information

Bioinspired trimesic acid anchored electrocatalysts with unique static and dynamic compatibility for enhanced water oxidation

Xiaojing Lin, Zhaojie Wang, Shoufu Cao, Yuying Hu, Siyuan Liu*, Xiaodong Chen, Hongyu Chen, Xingheng Zhang, Shuxian Wei, Hui Xu, Zhi Cheng, Qi Hou, Daofeng Sun, and Xiaoqing Lu**

X. Lin, Z. Wang, S. Cao, Y. Hu, S. Liu, X. Chen, X. Zhang, H. Xu, Z. Cheng, Q. Hou, D. Sun, X. Lu
School of Materials Science and Engineering,
China University of Petroleum,
Qingdao 266580, P. R. China
E-mail: wangzhaojie@upc.edu.cn; lsy@upc.edu.cn; luxq@upc.edu.cn

H. Chen, S. Wei
College of Science
China University of Petroleum,
Qingdao 266580, P. R. China

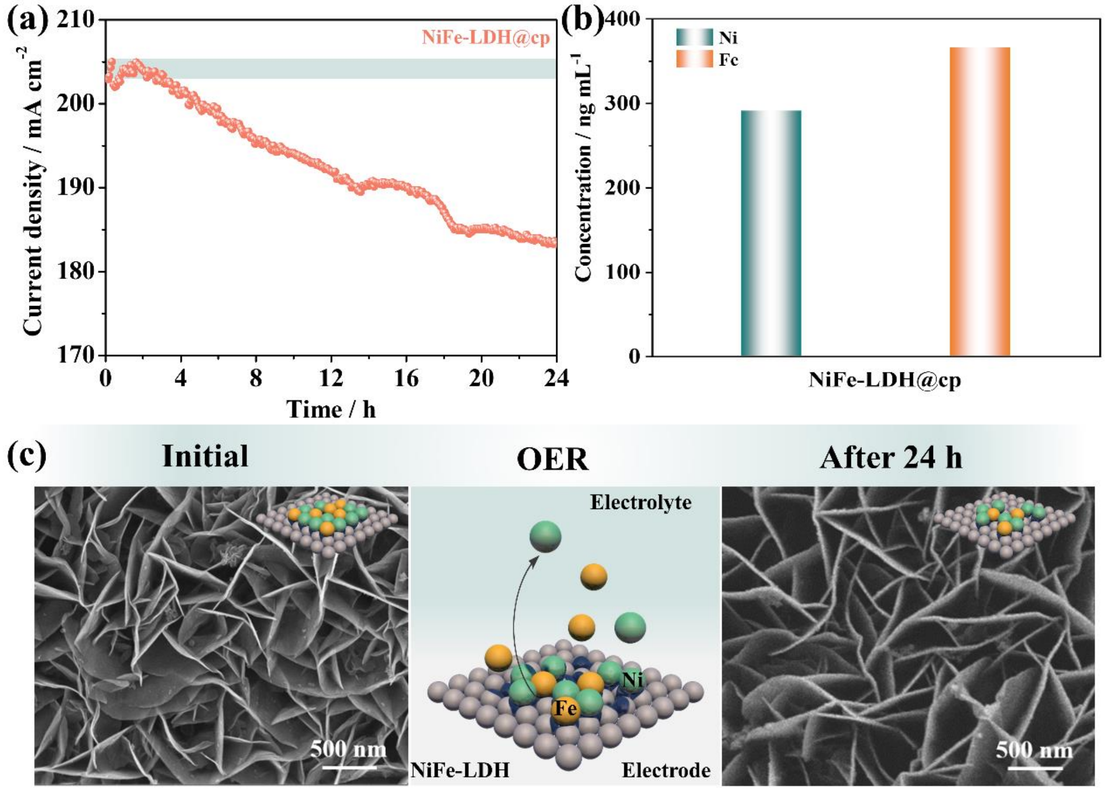

Figure S1. a) Chronopotentiometry curve of the NiFe-LDH@cp at 1.973V. **b)** Dissolved nickel and iron ions of NiFe LDH@cp detected by ICP-MS after electrolysis for 24 h. **c)** The morphology evolution of NiFe LDH@cp after electrolysis for 24 h.

In this research, the stability of initial NiFe-LDH@cp catalyst on carbon paper is tested using chronoamperometry (Figure S1a). Obviously, the current density decays rapidly from the beginning and only 90% was ined after 24 h (Figure S1a). In order to investigate the reason for the rapid deactivation of NiFe-LDH@cp, the concentration of metal ions in electrolyte and the evolution of catalyst morphology were explored after 24 hours of continuous electrolysis. The dissolution of Ni and Fe ions can be detected by inductively coupled plasma mass spectrometry (ICP-MS). As shown in Figure 1b and Table S1, 290.75 ng mL^{-1} Ni and 365.66 ng mL^{-1} Fe ions are dissolved in the electrolyte during water oxidation, which suggests that the metal segregation leads to catalysts deactivation. Then, the morphology was investigated by scanning electron microscopy (SEM). As shown in Figure S1c and Figure S2, the NiFe LDH shows nanoplates structure with a lateral size of ~594.87 nm. After electrolysis for 24 h, the morphology of catalyst was maintained, while partial ablation occurred at the edge and

surface of the NiFe-LDH nanoplates. The dissolution of LDH during OER process in alkaline solution is triggered by a local acidic environment caused by the slow diffusion of OH^{-} into the LDH interlayers.[1] On the other hand, dynamic metal dissolution-redeposition process in mixed-metal hydroxide will result in the phase segregation, accelerating the catalyst deactivation.[2] More serious leaching and dissolution of NiFe-LDH nanoplates will take place during the long-term industrial operation under large current densities. Consequently, we alternate the structure of LDH at the atomic scale to anchor mental atom and inhibit the leaching of transition metal hydroxide in alkaline media.

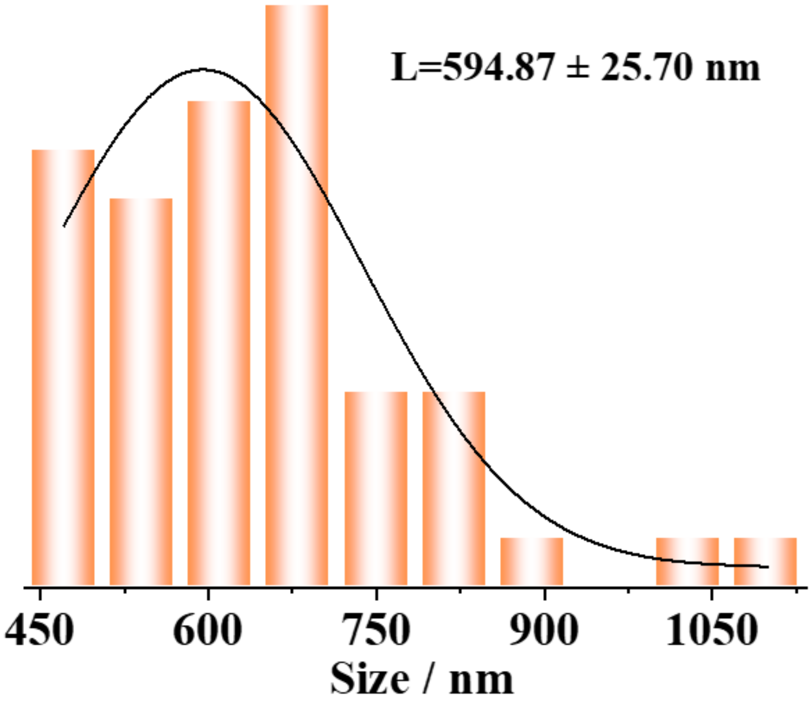

Figure S2. The particle size distribution of NiFe-LDH@cp.

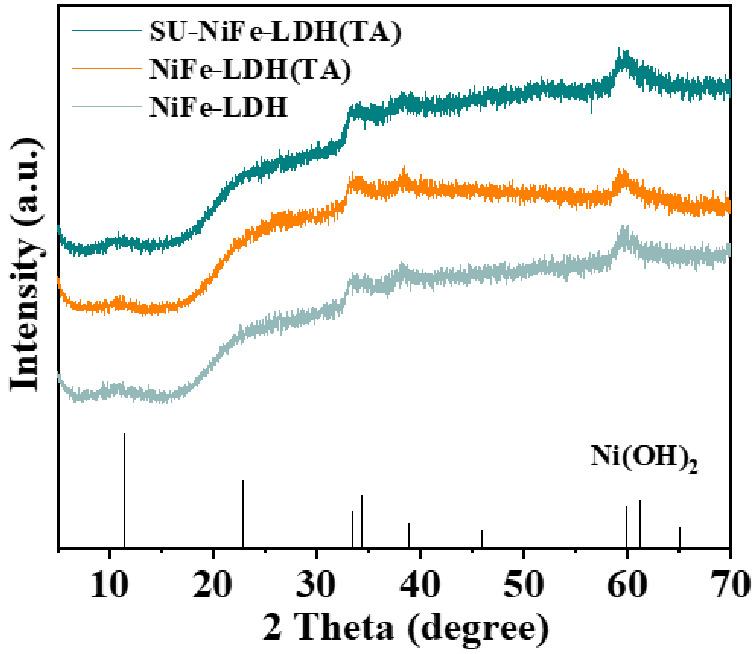

Figure S3. XRD patterns of NiFe-LDH, NiFe-LDH(TA), and SU-NiFe-LDH(TA).

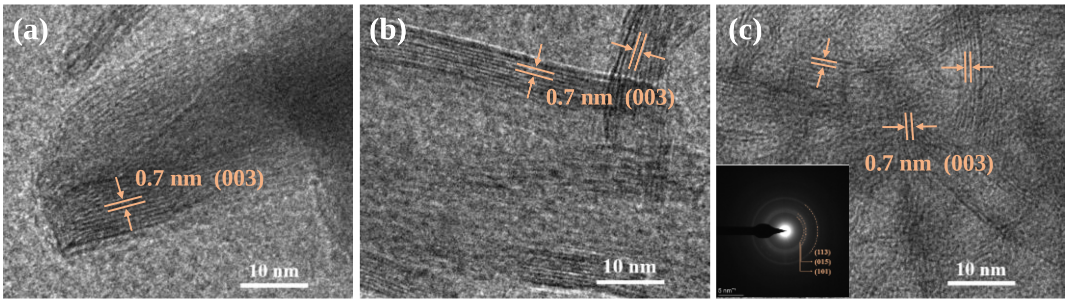

Figure S4. HRTEM images of **a)** NiFe-LDH@cp, **b)** NiFe-LDH(TA)@cp, and **c)** SU-NiFe-LDH(TA)@cp, and the inset present SAED pattern of SU-NiFe-LDH(TA)@cp.

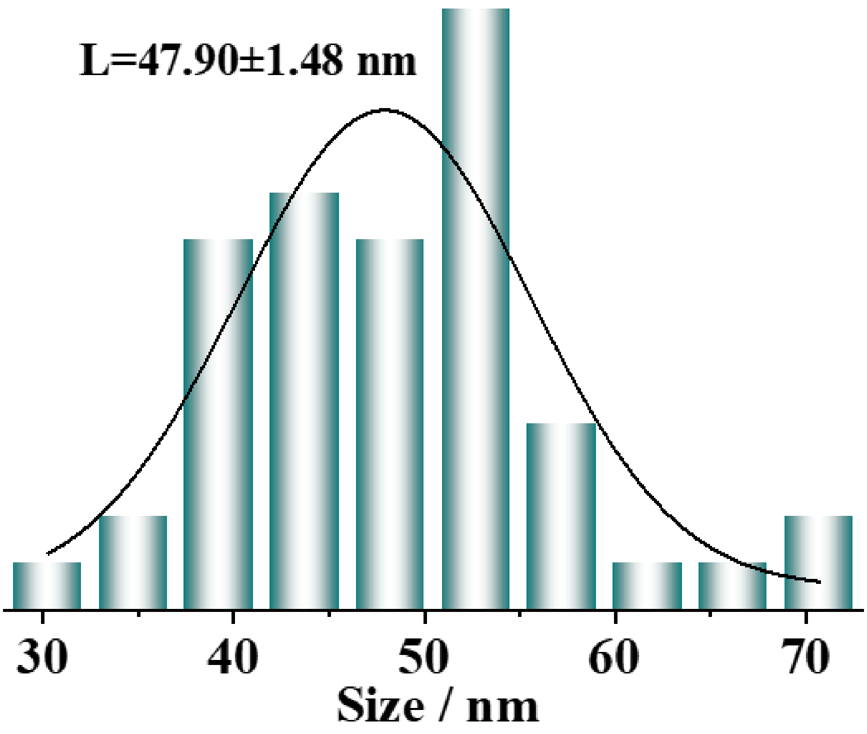

Figure S5. The particle size distribution of SU-NiFe-LDH(TA)@cp.

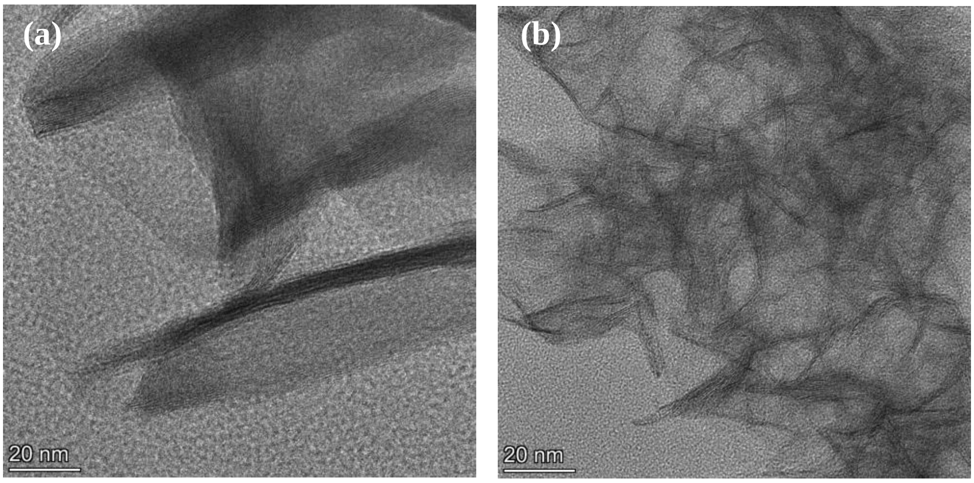

Figure S6. TEM images of **a)** NiFe-LDH@cp, and **b)** SU-NiFe-LDH(TA)@cp.

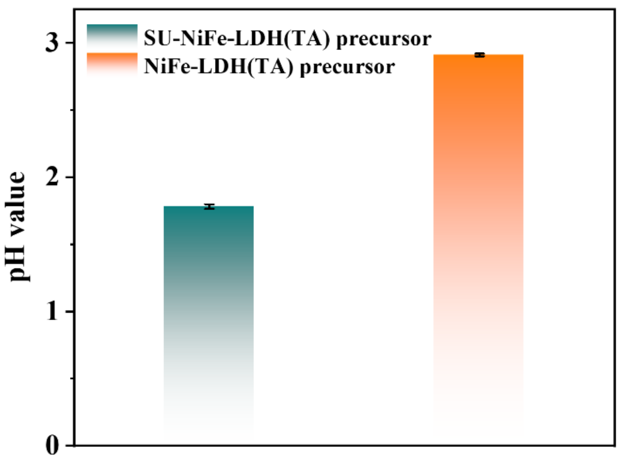

Figure S7. The pH value in the precursor solution of Fe^{2+} and Fe^{3+} for NiFe-LDH(TA) and SU-NiFe-LDH(TA), respectively.

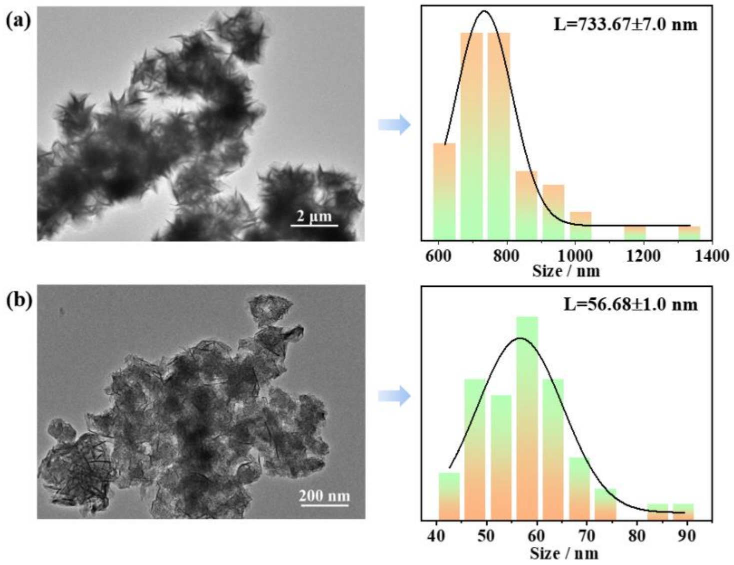

Figure S8. TEM image and the particle size distribution of **a)** NiFe-LDH(TA)@cp and **b)** SU-NiFe-LDH(TA)@cp.

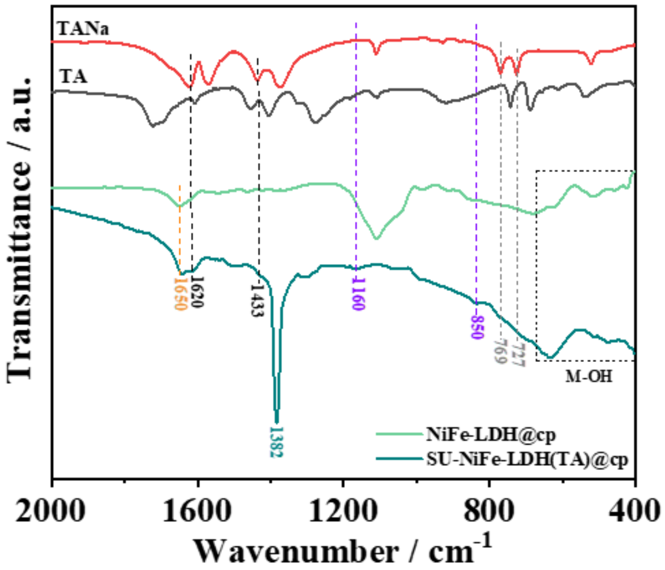

Figure S9. FTIR spectrum of NiFe-LDH@cp, SU-NiFe-LDH(TA)@cp, trimesic acid (TA) and 1,3,5-Benzenetricarboxylic acid, sodium salt (TANa).

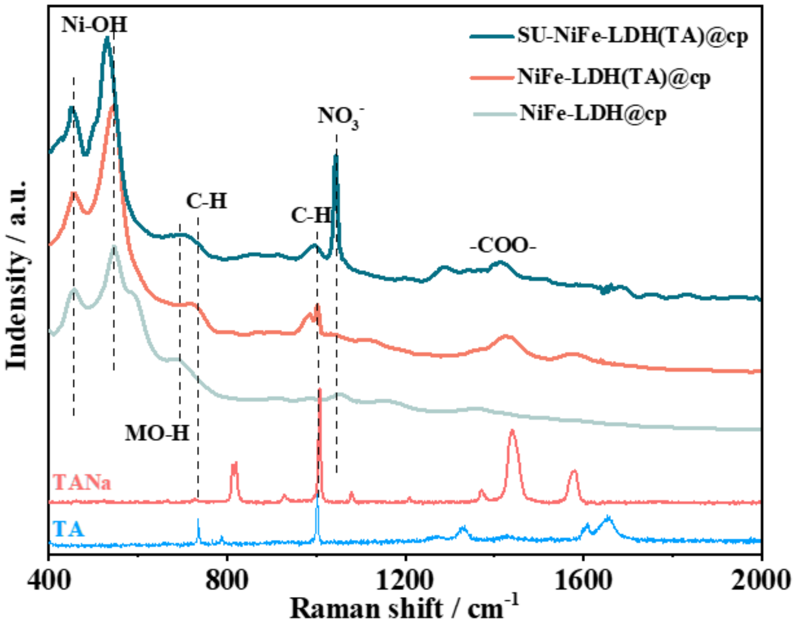

Figure S10. Raman spectra of trimesic acid (TA), 1,3,5-Benzenetricarboxylic acid, sodium salt (TANa), NiFe-LDH@cp, NiFe-LDH(TA)@cp, and SU-NiFe-LDH(TA)@cp.

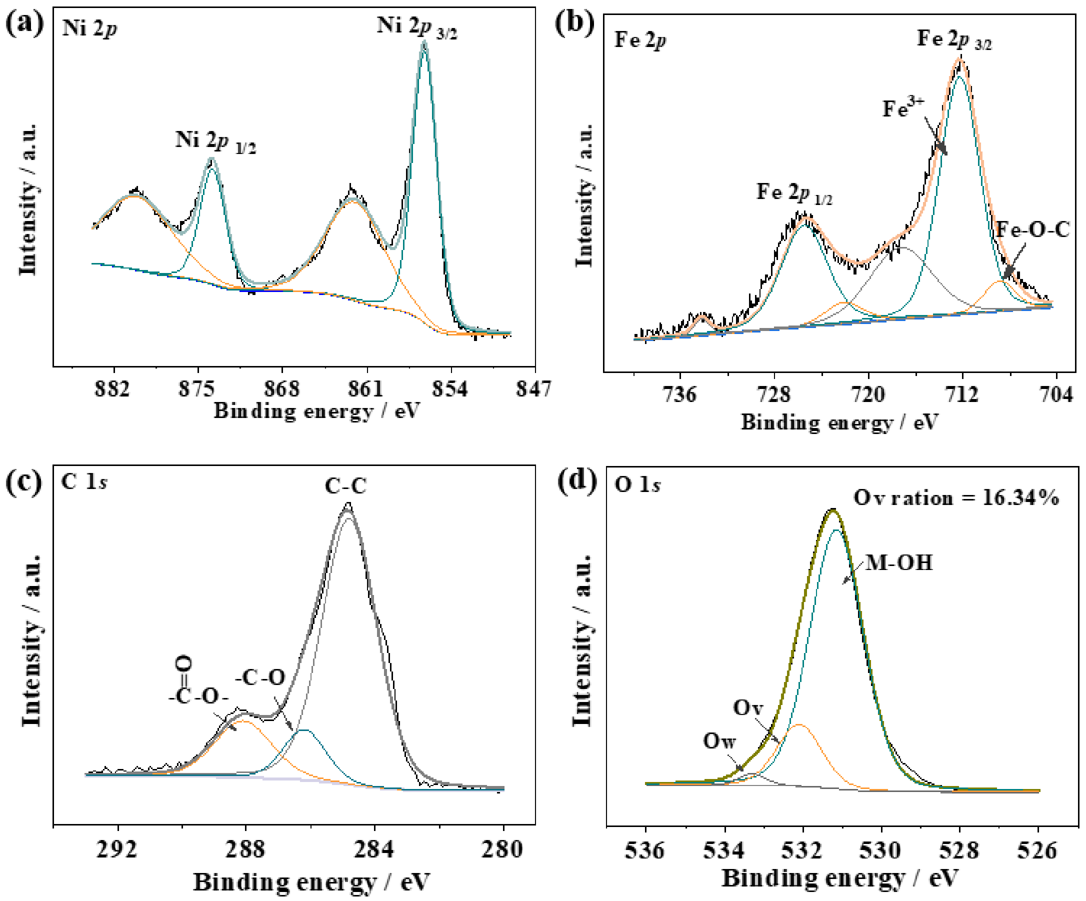

Figure S11. High resolution XPS spectrum of **a)** Ni 2*p*, **b)** Fe 2*p*, **c)** C 1*s*, and **d)** O 1*s* for NiFe-LDH(TA)@cp.

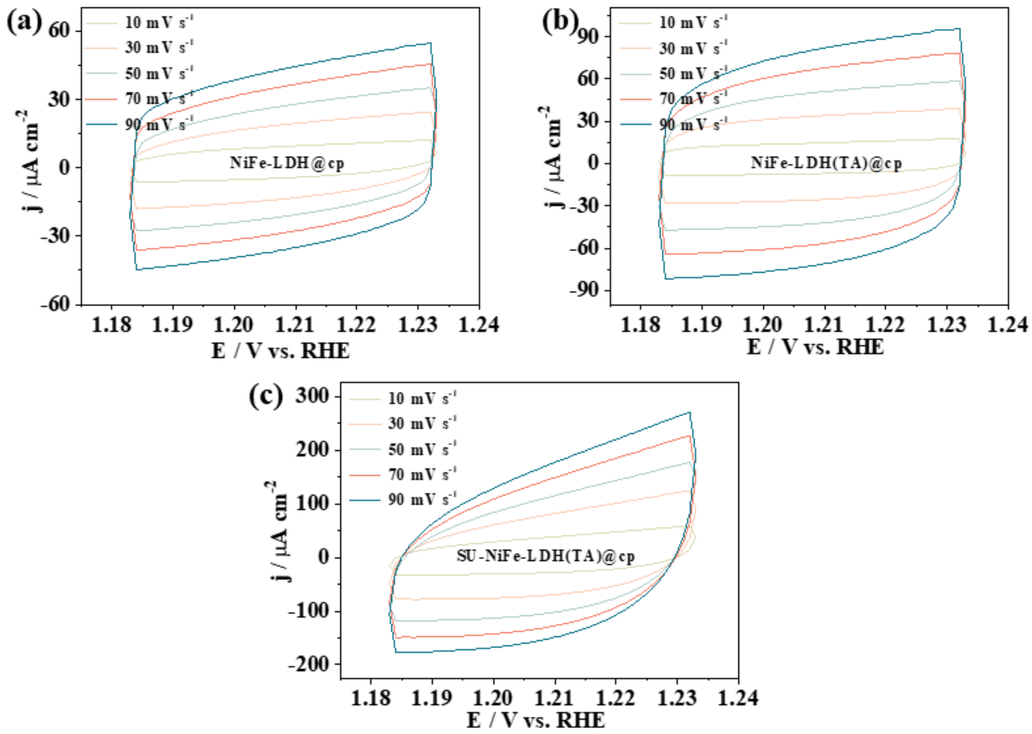

Figure S12. The CV curves of **a)** NiFe-LDH@cp, **b)** NiFe-LDH(TA)@cp, and **c)** SU-NiFe-LDH(TA)@cp at different scanning rates.

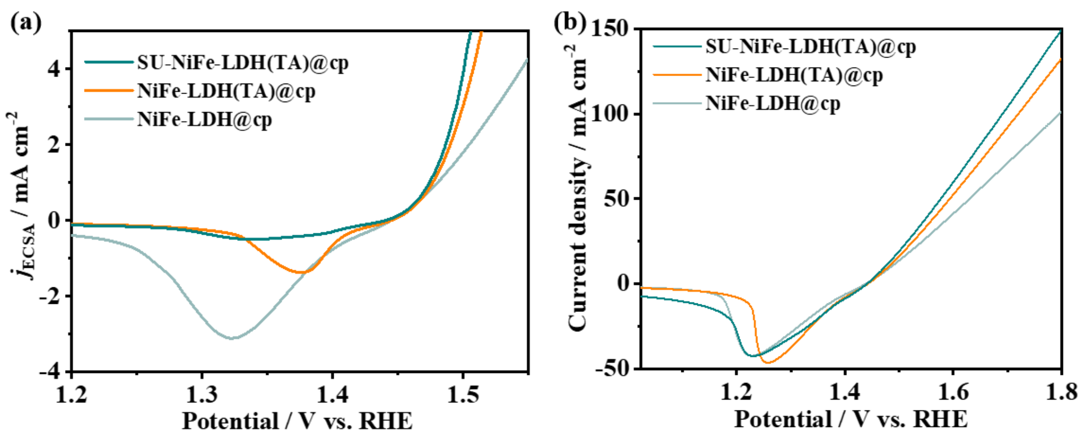

Figure S13. a) Intrinsic OER activity of the SU-NiFe-LDH(TA)@cp, NiFe-LDH(TA)@cp and NiFe-LDH@cp normalizing against ECSA. **b)** LSV curves of all samples without iR corrected.

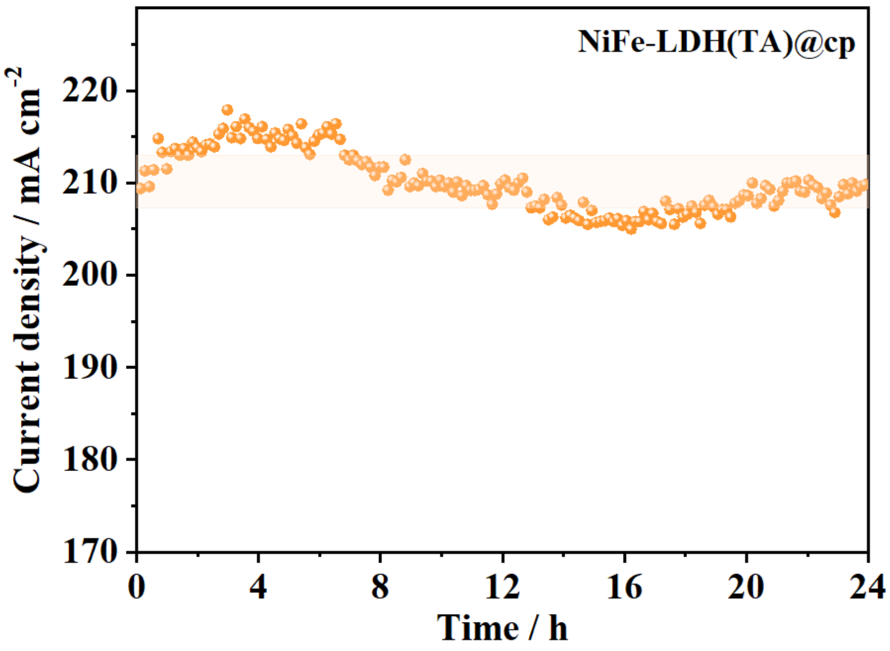

Figure S14. Chronoamperometric curve of NiFe-LDH(TA)@cp in 1M KOH.

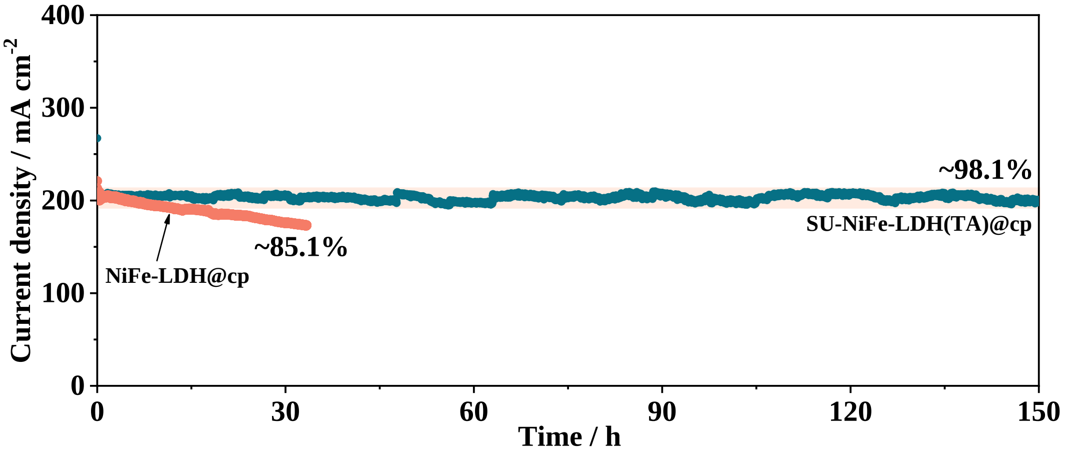

Figure S15. Comparison of Chronoamperometric curves for and SU-NiFe-LDH(TA)@cp in 1M KOH.

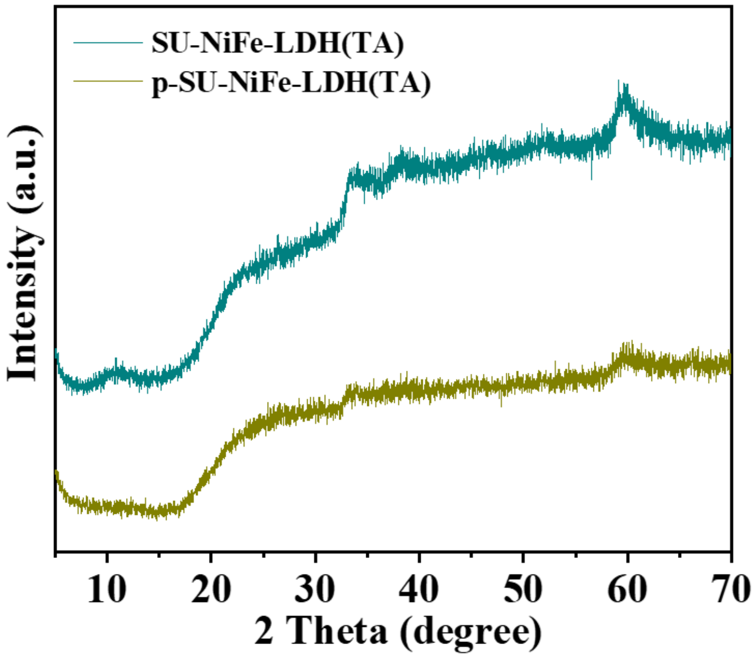

Figure S16. XRD of SU-NiFe-LDH(TA)@cp and p-SU-NiFe-LDH(TA)@cp.

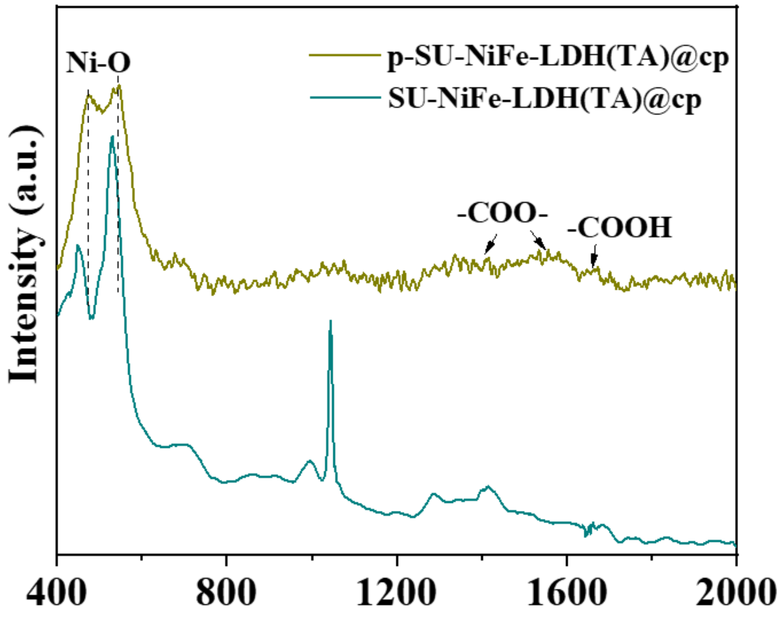

Figure S17. Raman spectra of SU-NiFe-LDH(TA)@cp and p-SU-NiFe-LDH(TA)@cp.

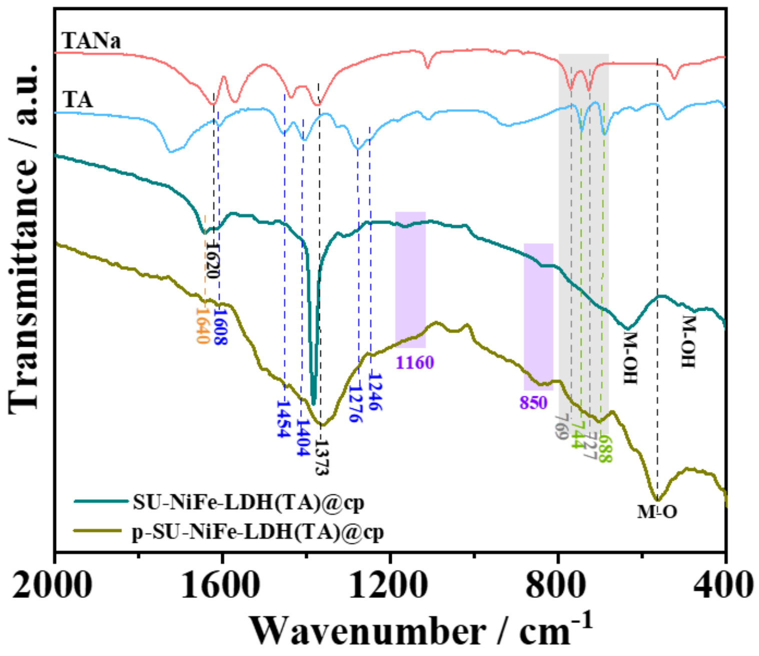

Figure S18. FTIR spectra of SU-NiFe-LDH(TA)@cp and p-SU-NiFe-LDH(TA)@cp.

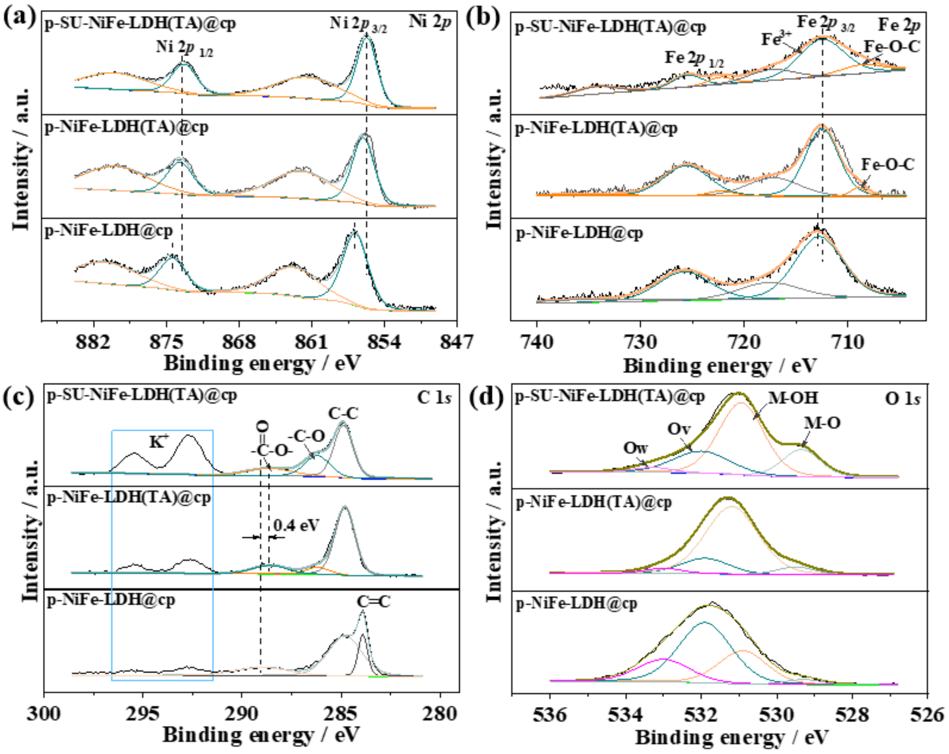

Figure S19. High resolution XPS spectrum of **a)** Ni 2*p*, **b)** Fe 2*p*, **c)** C 1*s*, and **d)** O 1*s* for p-SU-NiFe-LDH(TA)@cp, p-NiFe-LDH(TA)@cp and p-NiFe-LDH@cp. The C=C bond originates from the substrate of carbon paper

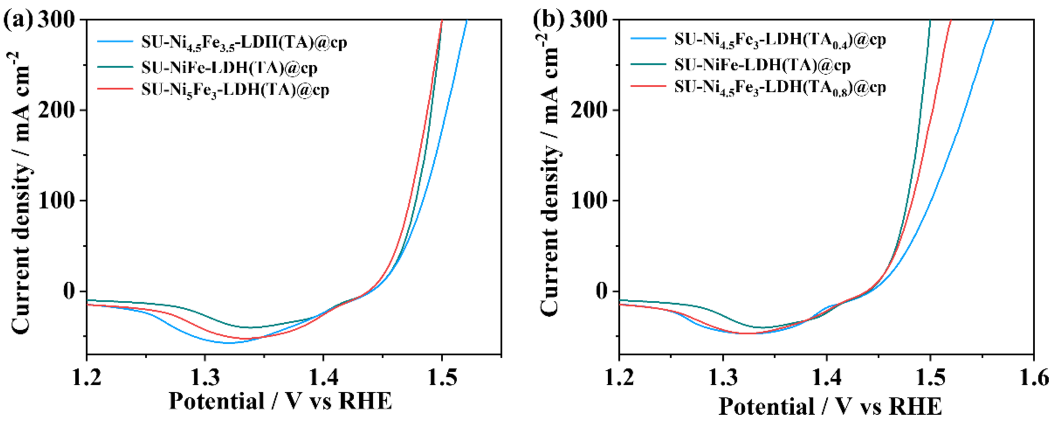

Figure S20. Linear sweep voltammetry of samples (SU-NixFey-LDH(TAz)@cp) with different dosages of the precursors: a) Different ratios of Ni and Fe precursors. b) Different dosages of trimesic acid in the precursors.

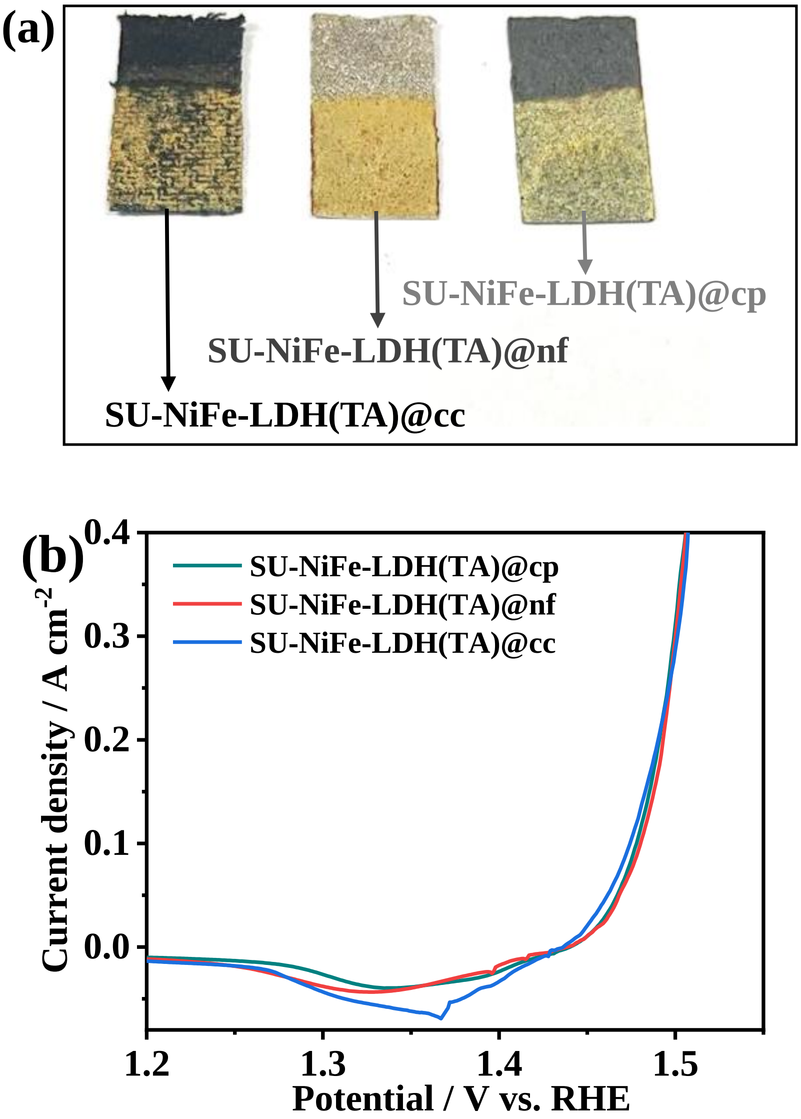

Figure S21. a) The photos of SU-NiFe-LDH(TA) on different basements, including carbon paper (cp), nickel foam (nf), and carbon cloth (cc). **b)** LSV curves of SU-NiFe-LDH(TA)@cp, SU-NiFe-LDH(TA)@nf, and SU-NiFe-LDH(TA)@cc with iR compensation.

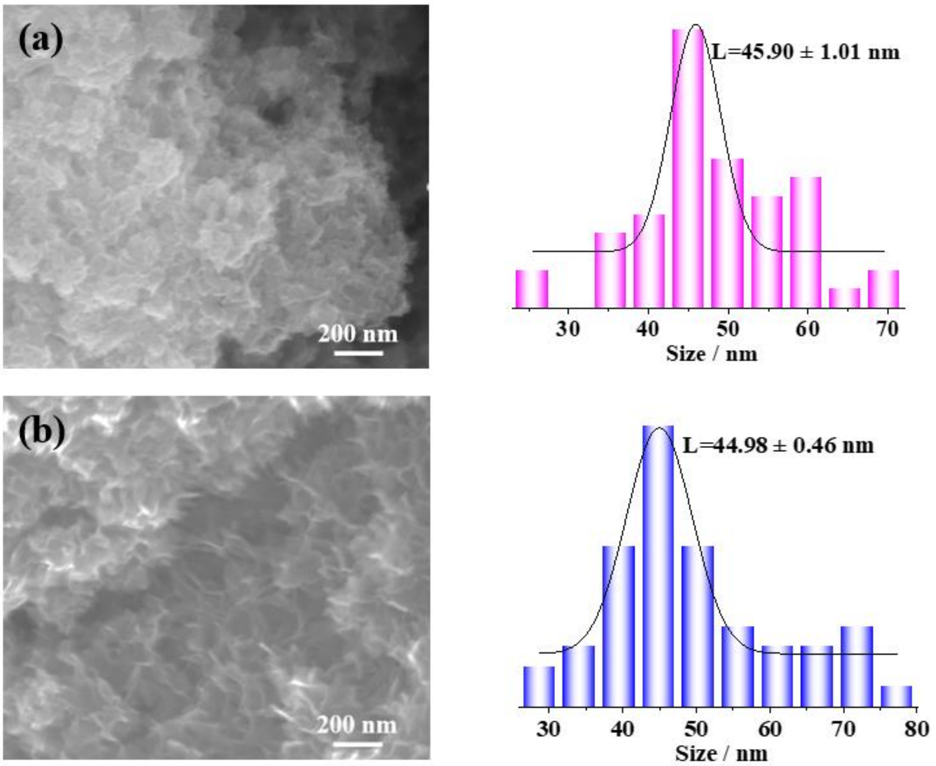

Figure S22. a) SEM image (left) and the particle size distribution (right) of SU-NiFe-LDH(TA)@nf. **b)** SEM image (left) and the particle size distribution (right) of SU-NiFe-LDH(TA)@nf after continuous operation for 1300 h at 1500 mA cm^{-2} current density.

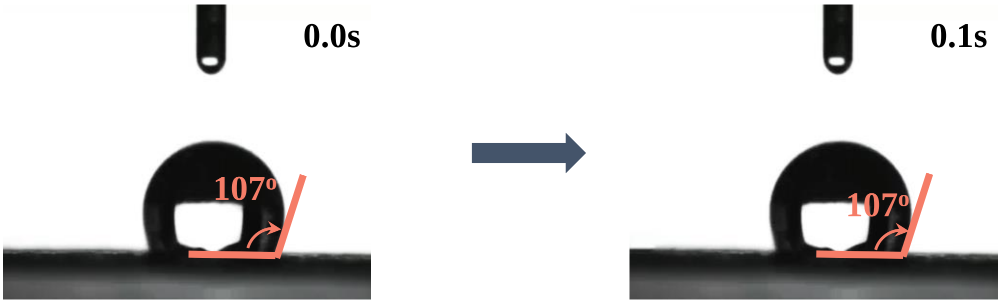

Figure S23. The contact angle of ultrapure water on carbon paper.

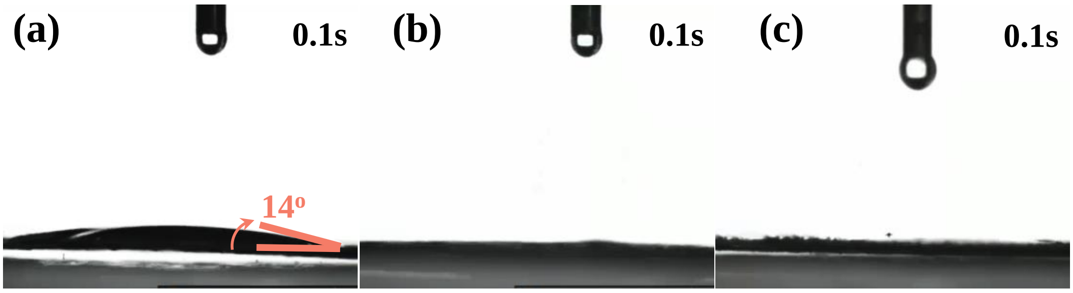

Figure S24. The contact angle of ultrapure water on **a)** NiFe LDH@cp, **b)** NiFe-LDH(TA)@cp, and **c)** SU-NiFe-LDH(TA)@cp after 0.1 s.

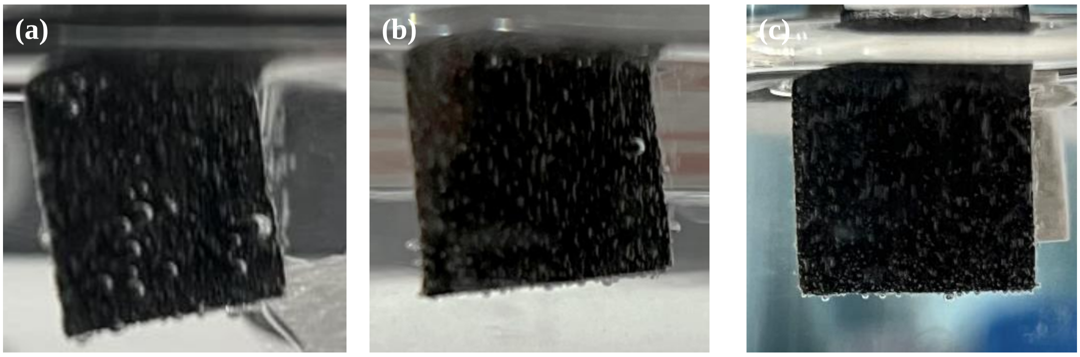

Figure S25. The digital photography of O_2 releasing behavior on **a)** NiFe LDH@cp, **b)** NiFe-LDH(TA)@cp, and **c)** SU-NiFe-LDH(TA)@cp at 200 mA cm^{-2}.

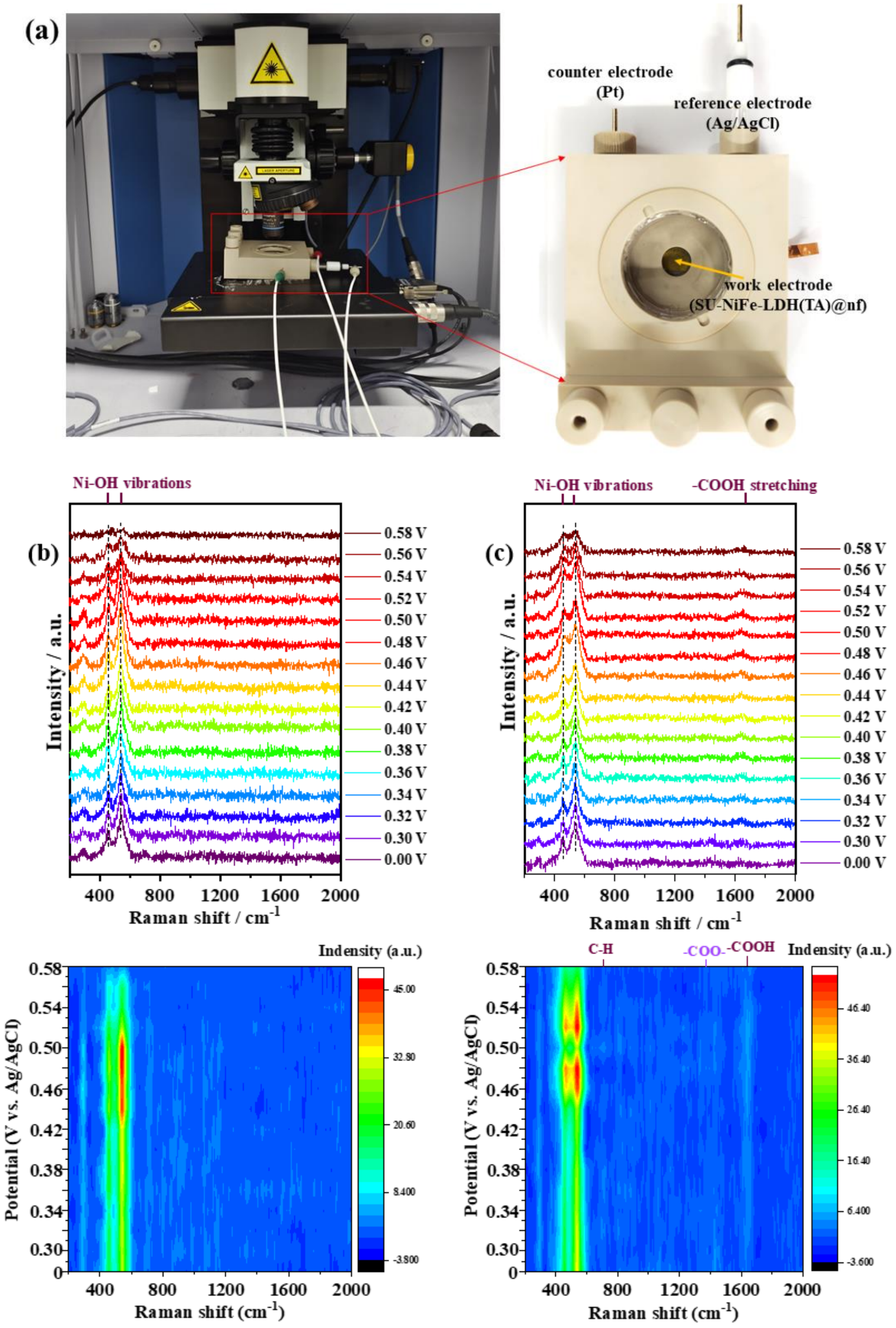

Figure S26. a) The in situ FTIR device**.** In situ Raman spectroscopy measurements on **b)** NiFe-LDH@cp and **c)** NiFe-LDH(TA)@cp sample at different applied potentials (V vs. Ag/AgCl).

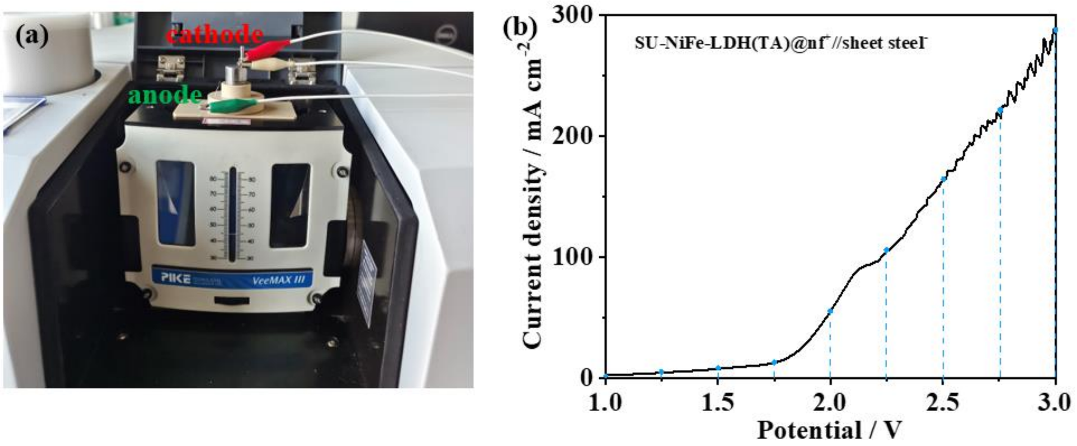

Figure S27. a) The in situ FTIR device assembled with SU-NiFe-LDH(TA)@nf as the anode and steel disc as the cathode and **b)** LSV curve of SU-NiFe-LDH(TA)@nf$^{(+)}$//steel disc$^{(-)}$.

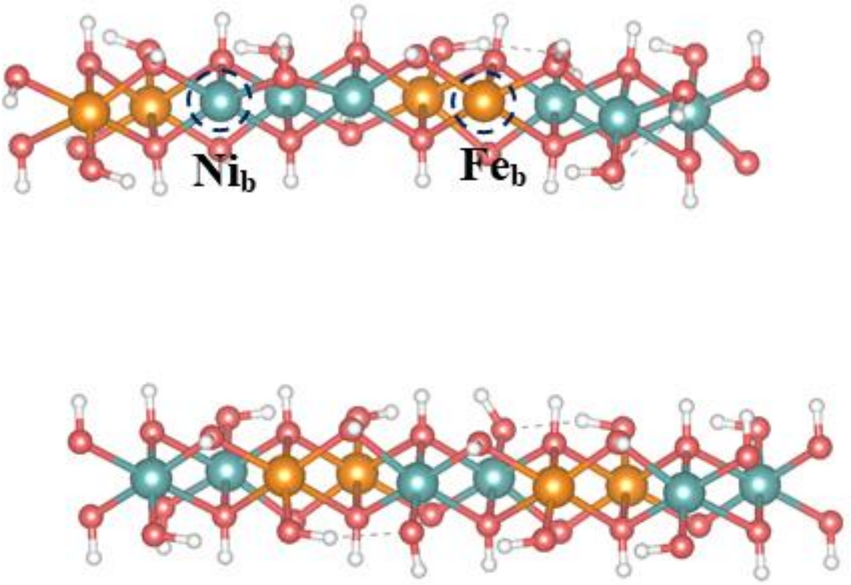

Figure S28. Model for NiFe-LDH. Green balls: Ni atoms; Orange balls: Fe atoms; Red balls: O atoms; Brown balls: C atoms; White atoms: H atoms.

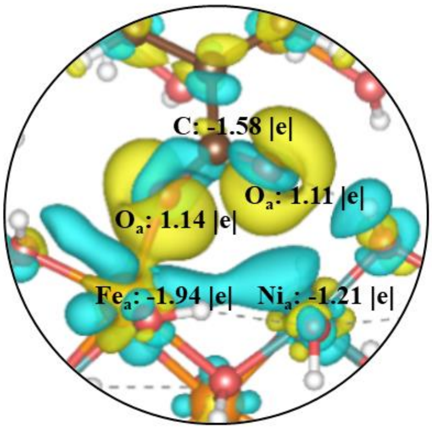

Figure S29. Bader charge at interface for SU-NiFe-LDH(TA).

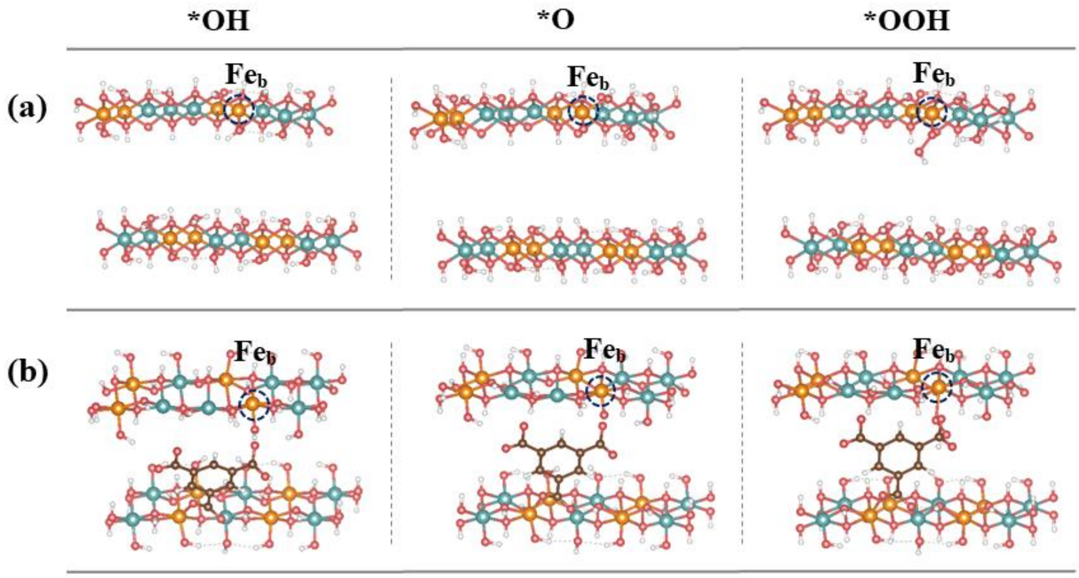

Figure S30. The optimized structures of *OH, *O, and *OOH adsorbed at Fe_b active site on **a)** NiFe-LDH and **b)** SU-NiFe-LDH(TA) models (Top view).

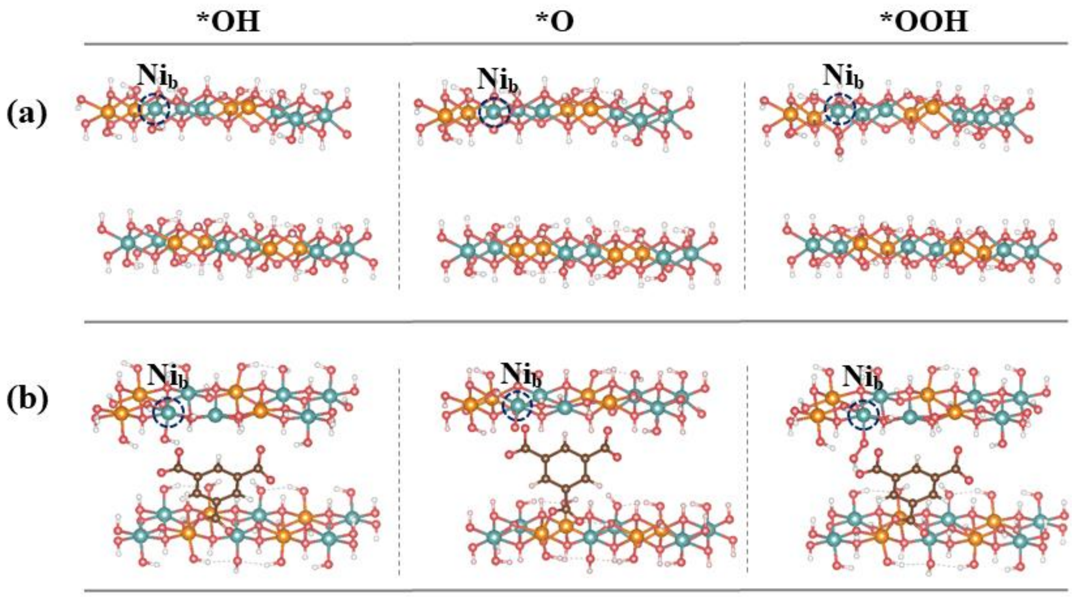

Figure S31. The optimized structures of *OH, *O, and *OOH adsorbed at Ni_b active site on **a)** NiFe-LDH and **b)** SU-NiFe-LDH(TA) models (Top view).

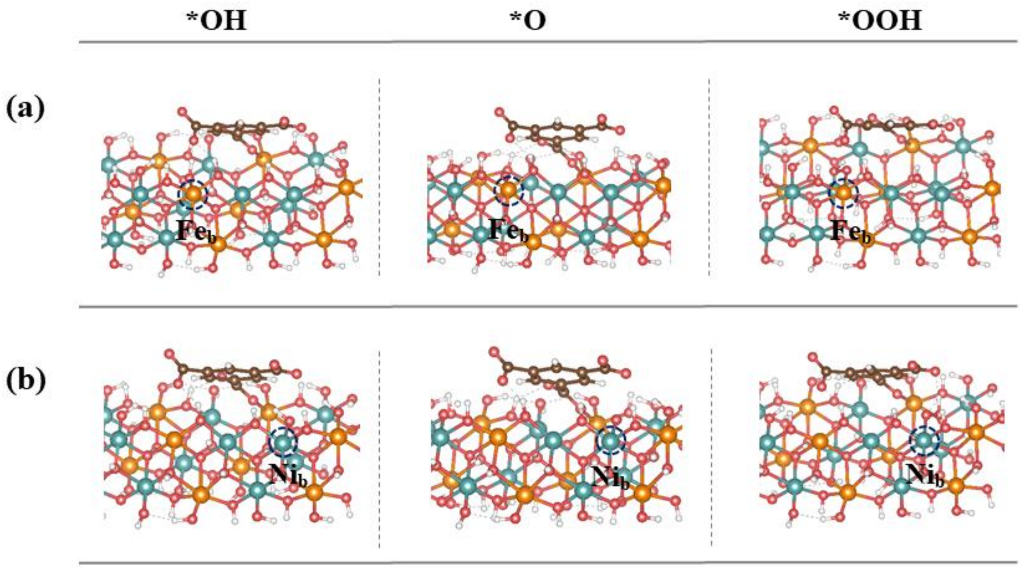

Figure S32. The optimized structures of *OH, *O, and *OOH adsorbed at **a)** Fe_b and **b)** Ni_b active site on SU-NiFe-LDH(TA) models (Side view).

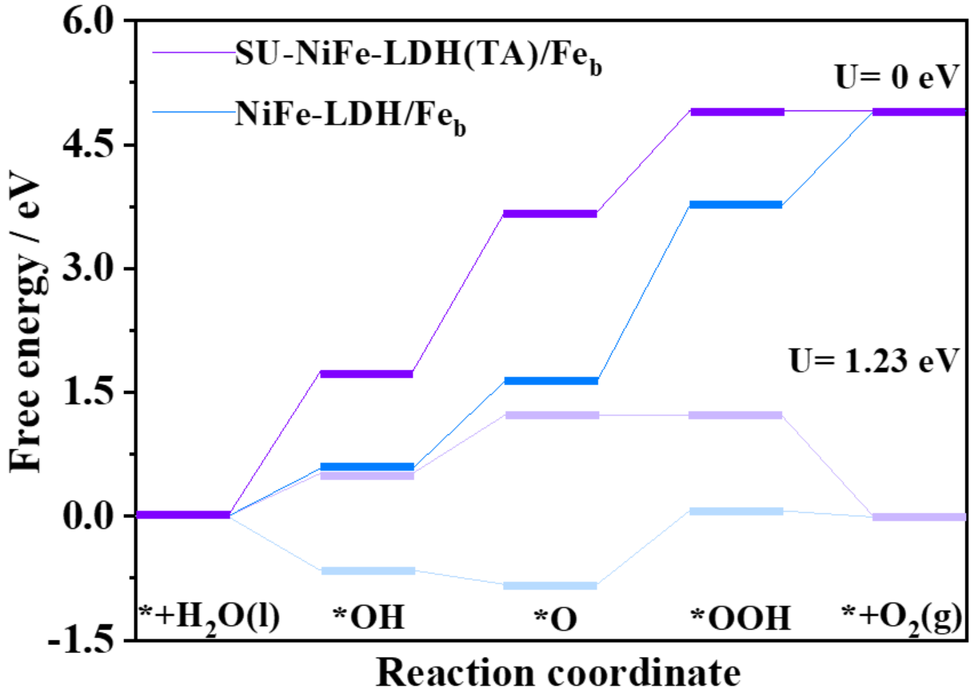

Figure S33. Free energy diagrams of NiFe-LDH and SU-NiFe-LDH(TA) models for Fe_b sites.

Table S1 Comparison of the OER performance and tafel between the SU-NiFe-LDH(TA) catalyst and other recently reported LDH-based OER electrocatalysts in 1 M KOH electrolyte.

Electrocatalysts	Substrate*	η at 10 mA cm^{-2}	Tafel / mV dec^{-1}
SU-NiFe-LDH(TA)	cp	219	31.1
NiFeCe-LDH@CP-3%[3]	cp	232	41.0
PM-NiFe-LDH/cp [4]	cp	230	47.0
NiFeW-4:1:1[5]	cp	249	68.0
$Fe_{0.4}Ni_{0.6}$LDH NPs[6]	cp	269	52.1
Co-Ni LDH[7]	cp	230	46.0
NiCo-LDH[8]	cp	367	40
Ni/Fe/CP[9]	cp	223	32.5
NiCo-LDHs@B_2O_3[10]	cp	213	61.0

* cp: carbon paper.

Table S2 EIS curves fitting parameters.

**Sample**	**Rs** (Ω cm^{-2})	**Error-$_{Rs}$** (%)	**Rct** (Ω cm^{-2})	**Error-$_{Rct}$** (%)
NiFe-LDH@cp	2.08	1.17	3.84	2.49
NiFe-LDH(TA)@cp	2.27	0.56	2.34	2.48
SU-NiFe-LDH(TA)@cp	2.12	0.49	1.69	4.44

Table S3 Peak positions of Ni 2p and Fe 2p for three samples before and after OER test and the relative content of oxygen defect by XPS

Samples	Peak positions of Ni element		Peak positions of Fe element		Ov (%)
	Ni $2p_{3/2}$	Ni $2p_{1/2}$	Fe $2p_{3/2}$	Fe $2p_{1/2}$	
NiFe-LDH@cp	856.1	873.7	712.5	725.5	16.54
NiFe-LDH(TA)@cp	856.1	873.7	712.5	725.5	16.34
SU-NiFe-LDH(TA)@cp	855.5	873.1	712.5	725.5	26.43
p-NiFe-LDH@cp	856.8	874.4	712.8	725.8	53.23
p-NiFe-LDH(TA)@cp	856.1	873.7	712.5	725.5	16.38
p-SU-NiFe-LDH(TA)@cp	855.5	873.1	712.5	725.5	26.58

Table S4 The Ni and Fe ions content in electrolyte for NiFe LDH@cp, NiFe-LDH(TA)@cp and SU-NiFe-LDH(TA)@cp samples after 24 h stability test at 200 mA cm^{-2}.

Working electrode	Testing time	Element content / ng mL^{-1}	
		Ni	Fe
NiFe-LDH@cp	24 h	290.75	365.66
NiFe-LDH(TA)@cp	24 h	20.71	18.21
SU-NiFe-LDH(TA)@cp	24 h	19.12	7.46

Table S5 Comparison of the stability between the SU-NiFe-LDH(TA) catalyst and other recently reported LDH-based OER electrocatalysts in 1 M KOH electrolyte.

Electrocatalysts	Substrate*	Current density / mA cm^{-2}	Time / h
SU-NiFe-LDH(TA)	nf	1500	1300
NiFe-LDH/NF-S-3h[11]	nf	500	150
LDH-Bir[12]	nf	100	160
P-V-NiFe LDH NSA[13]	nf	40	1000
Zn-(Ni/FeOOH) [14]	nf	1000	1000
Ni_xFe_{1-x}-AHNA [15]	nf	500	24

* nf: nickel foam.

Table S6 Comparison of the stability between the SU-NiFe-LDH(TA) catalyst and other recently reported advanced OER electrocatalysts in 1 M KOH electrolyte.

Electrocatalysts	Substrate*	Current density / mA cm^{-2}	Time / h
SU-NiFe-LDH(TA)	nf	1500	1300
$(NiCo)_3Se_4$ [16]	nf	1000	500
$Ni_3N@2M\text{-}MoS_2$ [17]	nf	1000	300
MnCoP [18]	nf	100	240
Ni_3S_2 [19]	nf	100	100
$Co_9S_8@Fe_3O_4$ [20]	nf	500	120

* nf: nickel foam.

Table S7 The O-H bond length in *OH and *OOH of NiFe-LDH and SU-NiFe-LDH(TA) models at different adsorption sites (Ni_b and Fe_b).

Catalysts	Adsorption sites	Intermediate	O–H bond length / Å
NiFe-LDH	Ni_b	*OH	0.979
		*OOH	0.982
	Fe_b	*OH	0.973
		*OOH	0.980
SU-NiFe-LDH(TA)	Ni_b	*OH	0.979
		*OOH	1.020
	Fe_b	*OH	1.043
		*OOH	0.992

Supplementary References:

1. Chen, R. et al. Layered structure causes bulk NiFe layered double hydroxide unstable in alkaline oxygen evolution reaction. *Adv. Mater.* **31**, e1903909 (2019).
2. Kuai, C. et al. Phase segregation reversibility in mixed-metal hydroxide water oxidation catalysts. *Nat. Catal.* **3**, 743-753 (2020).
3. Liao, Y. et al. Lattice distortion induced Ce-doped NiFe-LDH for efficient oxygen evolution. *Chem. Eng. J.* **464** (2023).
4. Zhang, X. et al. A simple synthetic strategy toward defect-rich porous monolayer NiFe-layered double hydroxide nanosheets for efficient electrocatalytic water oxidation. *Adv. Energy Mater.* **9**, 1900881 (2019).
5. Guo, P.-F. et al. Stable and active NiFeW layered double hydroxide for enhanced electrocatalytic oxygen evolution reaction. *Chem. Eng. J.* **426** (2021).
6. Ishizaki, M. et al. FeNi-layered double-hydroxide nanoflakes with potential for intrinsically high water-oxidation catalytic activity. *ACS Appl. Energy Mater.* **3**, 9040-9050 (2020).
7. Chen, C. et al. A bi-functional Co–Ni layered double hydroxide three-dimensional porous array electrode derived from ZIF-L(Co)@ZIF-L(Co, Ni) for oxygen evolution reaction and supercapacitors. *Int. J. Hydrogen Energy* **47**, 14896-14905 (2022).
8. Liang, H. et al. Hydrothermal continuous flow synthesis and exfoliation of NiCo layered double hydroxide nanosheets for enhanced oxygen evolution catalysis. *Nano Lett.* **15**, 1421-1427 (2015).
9. Xu, Z. et al. Coating of Ni on Fe (oxy)hydroxide: Superior catalytic activity for oxygen-involved reaction during water splitting. *ACS Sustain. Chem. Eng.* **7**, 19832-19838 (2019).
10. Sun, Z. et al. Amorphous boron oxide coated NiCo layered double hydroxide nanoarrays for highly efficient oxygen evolution reaction. *ACS Sustain. Chem. Eng.* **6**, 14257-14263 (2018).
11. Wan, Z. et al. Sulfur engineering on NiFe layered double hydroxide at ambient temperature for high current density oxygen evolution reaction. *ACS Appl.*

Energy Mater. **5**, 4603-4612 (2022).

12. Chen, Z. et al. TM LDH meets birnessite: A 2D-2D hybrid catalyst with long-term stability for water oxidation at industrial operating conditions. *Angew. Chem. Int. Ed. Engl.* **60**, 9699-9705 (2021).
13. Tang, Y. et al. Activating the hydrogen evolution and overall water splitting performance of NiFe LDH by cation doping and plasma reduction. *Appl. Catal. B: Environ.* **266**, 118627 (2020).
14. Zhang, X. et al. In situ reconstructed Zn doped $Fe_xNi_{(1-x)}OOH$ catalyst for efficient and ultrastable oxygen evolution reaction at high current densities. *Small*, e2203710 (2022).
15. Liang, C. et al. Exceptional performance of hierarchical Ni-Fe oxyhydroxide@NiFe alloy nanowire array electrocatalysts for large current density water splitting. *Energy Environ. Sci.* **13**, 86-95 (2020).
16. Abed, J. et al. In situ formation of nano Ni-Co oxyhydroxide enables water oxidation electrocatalysts durable at high current densities. *Adv. Mater.* **33**, e2103812 (2021).
17. Wu, T. et al. Engineering metallic heterostructure based on Ni_3N and 2M-MoS_2 for alkaline water electrolysis with industry-compatible current density and stability. *Adv. Mater.* **34**, e2108505 (2022).
18. Fu, W.-Y. et al. Sepaktakraw-like catalyst Mn-doped CoP enabling ultrastable electrocatalytic oxygen evolution at 100 $mA \cdot cm^{-2}$ in alkali media. *Rare Metals* **41**, 3069-3077 (2022).
19. Zhao, C.X. et al. An anionic regulation mechanism for structural reconstruction of sulfide electrocatalysts under oxygen evolution conditions. *Energy Environ. Sci.* **15**, 3257-3264 (2022).
20. Ji, Q. et al. Operando identification of active species and intermediates on sulfide interfaced by Fe_3O_4 for ultrastable alkaline oxygen evolution at large current density. *ACS Catal.* **12**, 4318-4326 (2022).
